# ICTV Virus Taxonomy Profile: *Pleolipoviridae*

**DOI:** 10.1099/jgv.0.000972

**Published:** 2017-11-10

**Authors:** Dennis H. Bamford, Maija K. Pietilä, Elina Roine, Nina S. Atanasova, Ana Dienstbier, Hanna M. Oksanen, ICTV Report Consortium

**Affiliations:** ^1^​ Department of Biosciences, University of Helsinki, Viikinkaari 9B, FI-00014, Helsinki, Finland; ^2^​ Department of Food and Environmental Sciences, University of Helsinki, Viikinkaari 9B, FI00014, Helsinki, Finland; ^3^​ Institute of Microbiology of the CAS, v.v.i., Videnska 1083, Prague, Czech Republic

**Keywords:** *Pleolipoviridae*, taxonomy, ICTV, Halorubrum pleomorphic virus 1

## Abstract

Members of the family *Pleolipoviridae* (termed pleolipoviruses) are pseudo-spherical and pleomorphic archaeal viruses. The enveloped virion is a simple membrane vesicle, which encloses different types of DNA genomes of approximately 7–16 kbp (or kilonucleotides). Typically, virions contain a single type of transmembrane (spike) protein at the envelope and a single type of membrane protein, which is embedded in the envelope and located in the internal side of the membrane. All viruses infect extremely halophilic archaea in the class Halobacteria (phylum Euryarchaeota). Pleolipoviruses have a narrow host range and a persistent, non-lytic life cycle. This is a summary of the International Committee on Taxonomy of Viruses (ICTV) Report on the taxonomy of the *Pleolipoviridae* which is available at www.ictv.global/report/pleolipoviridae.

## Virion

**Fig. 1.**
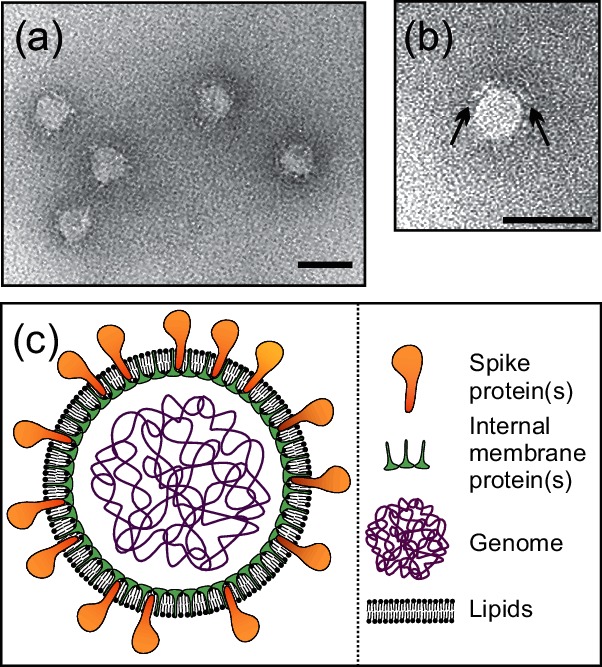
Morphology of pleolipovirus virions. (a) Electron micrograph of negatively stained Halorubrum pleomorphic virus 1 particles. (b) A close-up of one Halorubrum pleomorphic virus 1 virion. The arrows point to the spike structures covering the particle surface. Reproduced with permission from Pietilä *et al*. [5]. Scale bars in (a) and (b), 50 nm (c) Schematic presentation of the pleolipovirus virion.

Virions are enveloped pleomorphic membrane vesicles of 40–70 nm diameter (Table 1, Fig. 1a, b) with one or two types of major proteins forming spikes and one or two as internal membrane proteins (Fig. 1c). The spike and internal membrane proteins of Halorubrum pleomorphic virus 1 are VP4 and VP3 respectively. Virions lack a capsid or nucleocapsid.

**Table 1.** Characteristics of the family *Pleolipoviridae*

Typical member	Halorubrum pleomorphic virus 1 (FJ685651), species *Halorubrum virus HRPV1*, genus *Alphapleolipovirus*
Virion	Enveloped, pseudo-spherical and pleomorphic virions (diameters 40–70 nm), typically with a single type of spike protein at the envelope and a single type of internal membrane protein embedded in the envelope
Genome	Circular ssDNA, circular dsDNA or linear dsDNA, approximately 7–16 kilonucleotides or kbp
Replication	Possibly rolling-circle replication for circular molecules; protein-primed replication for linear molecules
Translation	Prokaryotic translation using viral mRNA and host ribosomes
Host range	Archaea, euryarchaeal *Halorubrum*, *Haloarcula* or *Halogeometricum* strains
Taxonomy	Three genera *Alphapleolipovirus*, *Betapleolipovirus* and *Gammapleolipovirus*

## Genome

**Fig. 2.**
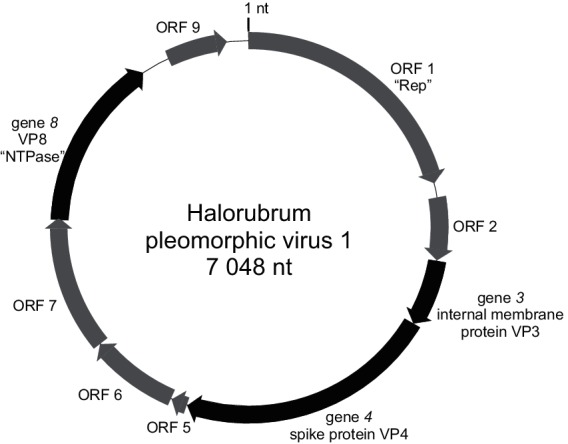
Genome organization of Halorubrum pleomorphic virus 1. Genes encoding structural proteins are coloured black, ORFs in grey. Rep, replication initiation protein; NTPase, nucleoside triphosphate hydrolase. The position of the first nucleotide (1 nt) is indicated.

Virus genomes are circular ssDNA of 7.0–10.7 kilonucleotides (Fig. 2), circular dsDNA of 8.1–9.7 kbp, or linear dsDNA of 16 kbp [1]. Members of the genus *Alphapleolipovirus* have circular ssDNA or dsDNA genomes, members of the genus *Betapleolipovirus* have circular dsDNA genomes with single-stranded discontinuities, and the only member of the genus *Gammapleolipovirus* has a linear dsDNA genome. A cluster of five genes/ORFs is conserved among the members of the family (Halorubrum pleomorphic virus 1 genes *3*, *4* and *8*, ORFs 6 and 7). The cluster includes genes encoding a spike and an internal membrane protein as well as an ORF encoding a putative NTPase. The only member of the genus *Gammapleolipovirus* is predicted to encode a putative type B DNA-dependent DNA polymerase. The genome ends bear terminal proteins.

## Replication

Pleolipoviruses are non-lytic and most likely enter cells by membrane fusion. Either rolling-circle or protein-primed replication may be used; transcription has not been studied. Progeny virions exit host cells continuously retarding host growth with concurrent unselective lipid acquisition implying that virions bud through the cell membrane.

## Taxonomy

### *Alphapleolipovirus* 

The genus includes the species *Haloarcula virus HHPV1*, *Haloarcula virus HHPV2*, *Halorubrum virus HRPV1*, *Halorubrum virus HRPV2* and *Halorubrum virus HRPV6.* The genomes of alphapleolipoviruses contain 8–15 ORFs, and besides the conserved gene/ORF cluster, an ORF encoding a putative rolling-circle replication protein and one ORF with unknown function [2–4]. Virions contain a single spike and internal membrane protein. Additionally, Halorubrum pleomorphic virus 1 virions contain one minor structural protein predicted to be an NTPase; tomographic reconstruction shows that the glycosylated spikes [5] form irregular arrays on the virion surface [6].

### *Betapleolipovirus* 

The genus includes the species *Halogeometricum virus HGPV1* and *Halorubrum virus HRPV3*. The betapleolipovirus genomes contain 12 or 15 ORFs. In addition to the cluster of five genes/ORFs, genomes share two ORFs encoding proteins with unknown function [4]. Virions contain one type of spike protein and one or two types of internal membrane proteins. The spike protein of Halogeometricum pleomorphic virus 1 is lipid-modified [6].

### *Gammapleolipovirus* 

*Haloarcula virus His2* is the only species of this genus. The member has a genome with 35 ORFs [7] and a virion consisting of one type of internal membrane protein and two types of spike protein as well as one minor structural protein [6]. One of the spike proteins is lipid-modified.

## Resources

Full ICTV Online (10th) Report: www.ictv.global/report/pleolipoviridae.
